# The effects of targeted vagus nerve stimulation on glucose homeostasis in STZ-induced diabetic rodents

**DOI:** 10.3389/fnins.2023.1179276

**Published:** 2023-06-15

**Authors:** Elliott W. Dirr, Yogi Patel, Richard D. Johnson, Kevin J. Otto

**Affiliations:** ^1^J. Crayton Pruitt Family Department of Biomedical Engineering, University of Florida, Gainesville, FL, United States; ^2^Department of Biomedical Engineering, Georgia Institute of Technology, Atlanta, GA, United States; ^3^Department of Neuroscience, University of Florida, Gainesville, FL, United States; ^4^Department of Physiological Sciences, University of Florida, Gainesville, FL, United States; ^5^Department of Neurology, University of Florida, Gainesville, FL, United States; ^6^Department of Materials Science and Engineering, University of Florida, Gainesville, FL, United States; ^7^Department of Electrical and Computer Engineering, University of Florida, Gainesville, FL, United States

**Keywords:** diabetes, bioelectronic medicine, neuromodulation, pancreatic, insulin

## Abstract

During type 1 diabetes, an autoimmune attack destroys pancreatic β-cells leading to the inability to maintain glucose homeostasis. These β-cells are neuroresponsive endocrine cells which normally secrete insulin partially in response to input from the vagus nerve. This neural pathway can be utilized as a point of therapeutic intervention by delivering exogenous stimulation to drive increased insulin secretion. In this study, a cuff electrode was implanted on the pancreatic branch of the vagus nerve just prior to pancreatic insertion in rats, and a continuous glucose meter was implanted into the descending aorta. Streptozotocin (STZ) was used to induce a diabetic state, and changes in blood glucose were assessed using various stimulation parameters. Stimulation driven changes in hormone secretion, pancreatic blood flow, and islet cell populations were assessed. We found increased changes in the rate of blood glucose change during stimulation which subsided after stimulation ended paired with increased concentration of circulating insulin. We did not observe increased pancreatic perfusion, which suggests that the modulation of blood glucose was due to the activation of b-cells rather than changes in the extra-organ transport of insulin. Pancreatic neuromodulation showed potentially protective effects by reducing deficits in islet diameter, and ameliorating insulin loss after STZ treatment.

## Introduction

Bioelectronic medicine is an emerging field that aims to replace traditional pharmacological therapies by utilizing electrical neuromodulation rather than medications (Olofsson and Tracey, 2017). Treating disease using neuromodulation relies on an understanding and exploitation of neural pathways which modulate organ function through the innervation of specific effector cells. This paradigm allows patients to take over control of subconscious physiological processes by applying exogenous electrical stimulation to the autonomic nervous system. This, in turn, drives neurotransmitter release at the desired neuroresponsive effector cell.

One common bioelectronic therapeutic target is the vagus nerve. The vagus nerve provides afferent and efferent signaling to the heart, lungs, and most of the visceral organs in the abdominal cavity including the pancreas (Evans and Murray, 1954; Agostoni et al., 1957; Prechtl and Powley, 1990). Devices for vagus nerve stimulation have provided safe and effective ways to modulate neural circuitry that has been implicated in epilepsy (Englot et al., 2011), headache (Straube et al., 2015), depression (Nemeroff et al., 2006), and obesity (Johannessen et al., 2017). These therapies have utilized FDA-approved devices since 1997 and are well-tolerated (Schachter, 2002).

Type 1 diabetes (T1D) is a disease characterized by an autoimmune attack that slowly destroys pancreatic β-cells resulting in the loss of homeostasis and induction of a hyperglycemic state (Eisenbarth, 1968; Atkinson, 2012). As of 2020, over 1.6 million Americans have T1D, and there is currently no cure for the disease. Treatment of T1D relies on the patient manualizing the task of the β-cells through blood glucose testing and exogenous insulin injection (Diabetes Control Complications Trial Research Group et al., 1993; Hirsch, 1996). While these treatments can be effective, the therapy is not only dependent on patient-specific drug efficacy but also compliance and availability of medication. Furthermore, due to the dynamic nature of metabolism, patients are tasked with a continuous treatment regimen. This leads to an enormous economic burden and negatively affects a patient's quality of life. Recent advances have been made which can lessen the patient burden by integrating sensing and insulin delivery into one closed-loop device; however, these approaches still require a constant supply of exogenous insulin (Messer et al., 2018).

The ability to control innate β-cells has large practicality in the development of closed-loop therapies for T1D. While the progression of T1D is shaped by a chronic autoimmune attack on β-cells, functional cells have been observed in patients decades after disease onset (Matveyenko and Butler, 2008; Keenan et al., 2010). A large body of work has demonstrated that the electrical stimulation of the cervical and subdiaphragmatic trunks of the vagus nerve results in the modulation of blood glucose, presumably through M_3_ muscarinic acetylcholine receptor-mediated release of insulin from β-cells (Verspohl et al., 1990; Gautam et al., 2006; Meyers et al., 2016; Dirr et al., 2020; Güemes Gonzalez et al., 2020). A recent study has shown that subdiaphragmatic stimulation results in a decrease in the glucose excursion during oral glucose tolerance testing in a model of T2D (Yin et al., 2019). While these studies provide important insight toward creating neuromodulatory-based therapy, the mechanism has been obfuscated by using untargeted stimulation.

This study aimed to evaluate the use of targeted pancreatic vagus nerve stimulation (pVNS) to control blood glucose in a diabetic model. In the following experiments, stimulation was applied to a branch of the vagus nerve which exclusively innervates the pancreas. This approach eliminates the confounding effects that gastric motility, nutrient absorption, and modulation of liver function have on blood glucose concentration.

## Methods

### Surgical implantation

All procedures involving animals were approved by the University of Florida Institutional Animal Care and Use Committee (IACUC) as well as the Animal Care and Use Review Office (ACURO). All studies presented in this study were completed using male Lewis rats housed on a 12-h reverse light–dark cycle. Rats weighing ~250–300 g were anesthetized using 3% isoflurane and given 5 mg/kg meloxicam. The duration of surgery ranged from 3.6 to 4.9 h (mean = 3.9). Under aseptic conditions, a midline abdominal incision was made, and using a Zeiss OPMI-1FC variable zoom dissection microscope, the pancreatic branch of the vagus nerve (~150 μm) was identified using anatomic landmarks as previously reported (Dirr et al., 2019). The identity of this small-diameter nerve was previously determined by recording compound neural action potentials after the electrical stimulation of the subdiaphragmatic vagus trunk (Dirr et al., 2019). A 300-μm inner diameter cuff electrode with two stainless steel electrodes (Microprobes for Life Sciences, Gaithersburg, MD, USA) was placed around the nerve and electrically isolated using a two-part silicone (World Precision Instruments, Sarasota, FL, USA). A continuous glucose monitor (CGM) was then placed into the descending aorta just proximal to the aortic bifurcation according to the manufacturer's recommendations (Data Sciences International, St. Paul MN, USA). The electrode leads were then routed subcutaneously to the skull. Three titanium screws were used as anchors in the skull, and a Delrin barrel (Plastics1, Roanoke, VA, USA) encased in dental cement (Lang Dental, Wheeling IL, USA) was used to secure the connector pins for the electrode leads. The midline incision was closed using wound clips. After recovery, the animals were given meloxicam for 4 days post-surgery and buprenorphine for up to 2 days post-surgery.

### Continuous glucose monitor maintenance

Telemetric CGMs were initially calibrated using a two-point calibration curve generated from an oral glucose tolerance test as recommended by the manufacturer. The glucose tolerance test was administered prior to diabetic induction for animal welfare. After at least 6 h of fasting, blood glucose was sampled from the tail vein using a hand-held blood glucose meter (Bayer, Leverkusen, Germany). After baseline measurement, 5 g/kg dextrose was administered by oral gavage. The current generated by the CGM was monitored until the peak was observed, and the tail blood was once again sampled 3 min after the peak and tested for blood glucose concentration. An equation was generated for each CGM to convert current to blood glucose concentration. To account for sensor degradation, a single blood glucose measurement was taken once per week from the tail using the same hand-held glucometer. These data were used to adjust the Y-intercept of each CGM's calibration equation.

### Diabetic induction

Animals recovered after surgery until their weight stabilized and returned to pre-surgical values. A diabetic state was induced through streptozotocin (STZ) injections (Dirr et al., 2019). In short, two 65 mg/kg intraperitoneal injections of STZ (Sigma-Aldrich, St. Louis, MO, USA) were given separated by 72 h. The glycemic state was evaluated 48 h after the second injection, and subjects with a blood glucose concentration over 300 mg/dl were classified as successfully induced to a hyperglycemic state. Animals with blood glucose below 300 mg/dl were excluded from the study. After diabetic induction, animals received five units of Lantus insulin per day following the last stimulation session of each day.

### Electrical stimulation

**Figure 1:**
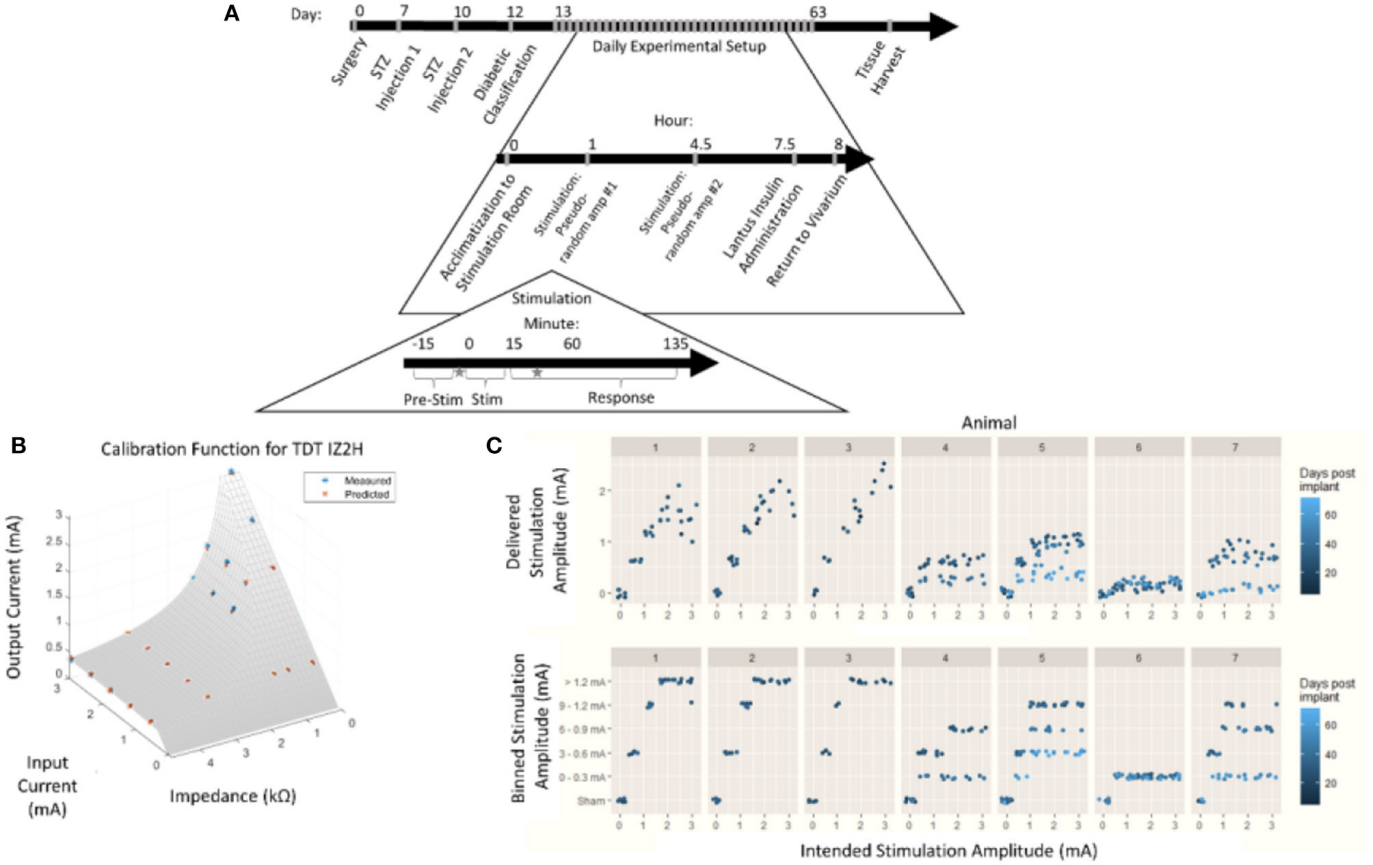
Chronic pancreatic neuromodulation experimental design. **(A)** Timeline of pancreatic neuromodulation experimental design. One week after surgery, rodents received chemical induction of T1D using two i.p. doses of STZ separated by 72 h. Forty-eight hours after the second injection, rodents with blood glucose >300 mg/dl were classified as diabetic and used further in this study. Rodents received two stimulation sessions separated by 4 h per day for up to 36 days. Times at which blood was collected for hormone measurement are denoted by a star. **(B)** Model used to determine delivered current delivered in each stimulation session from the intended current requested. **(C)** Distribution of intended and delivered stimulus amplitude over time for each animal, and stimulation amplitude bins.

The amplitude of stimulation for a given session was a value chosen from the set: 3.0, 2.4, 1.8, 1.2, 0.6, and 0 mA (“sham” stimulation). Prior to the initiation of the stimulation, a schedule for stimulation amplitude was set for each animal (*n* = 7) determined by sampling without replacement from the amplitude set above. Thus, any given amplitude did not repeat until the entire set was completed. These were delivered via pVNS and administered up to twice per day during the animal's dark cycle according to Figure 1. Throughout the course of the study, subjects were examined during stimulation for current leak and subsequent recruitment of abdominal muscles. Any subject displaying indications of electrode dysfunction was removed from the study, and the data are not included in this study. Off-target effects on other visceral organs were not evaluated.

During an individual stimulation session, the animals were moved from the vivarium to the stimulation room and given at least 1 h to acclimate. Blood glucose concentration was continuously measured, and average values were recorded once every 10 s. After an initial acclimatization period, subjects were individually plugged into an IZ2H Stimulator (Tucker-Davis Technologies, Alachua, FL) and administered current-controlled, cathode-leading, charge-balanced, biphasic stimulation with various waveforms. During the first stimulation sessions of the day, bipolar pulses were with the cathode-leading pole on the caudal electrode and the return on the rostral electrode. The second stimulation session reversed the polarity, resulting in cathode-leading pulses being applied to the rostral electrode. Each stimulation session consisted of the selected amplitude pulses (800 μs per phase) repeated at 10 Hz for 15 min. Amplitudes were not repeated within anode- or cathode-leading conditions until the complete set of amplitudes was tested. Each amplitude was applied at least three times. Subsequent stimulations were given at least 4 h after the first daily stimulation. Animals were returned to the vivarium 4 h after the last stimulation session of each day.

Due to the impedance of the electrodes and the 15 V compliance of the IZ2H Stimulator, the true current delivered was found to be lower than the intended current. The discrepancy between the delivered current and the intended current required a *post-hoc* recalibration curve. To build the *post-hoc* calibration curve, previously mentioned stimulation amplitudes were delivered through a range of known resistive loads between 2.2 and 47.9 kΩ. The voltage drop across the load was measured, and the delivered current was calculated. An equation was fit to the delivered current as a function of the intended current and electrode impedance (Figure 1B) empirically. The following equation was used to correct stimulus amplitude:


(1) $$\begin{array}{l} i_{out}=7383\;*\;{\left({z\;*\;i}_{in}\right)}^{-0.9234}\;*\;i_{in} \end{array}$$

where *z* represented electrode impedance (kΩ)*, i*_*in*_represented intended stimulus amplitude (mA), and *i*_*out*_ represented *post-hoc* corrected delivered amplitude (mA).

Stimulation amplitudes were adjusted from the intended current to the delivered current based on this equation (Figure 1C, top). The continuous variable “delivered current” was then discretized by re-binning into the original stimulation amplitude ranges (Figure 1C, bottom). This allowed for the assessment of effects between stimulation amplitudes with a limited sample size.

### Analysis of blood glucose modulation

Analysis of blood glucose data was completed using custom MATLAB Scripting. Blood glucose measures were down-sampled to one measure per minute. Preceding each stimulation, a 15-min baseline collection of blood glucose concentration served as a normalization period. A 95% confidence interval was constructed for each trial, and continuous blood glucose measurements were normalized to the mean blood glucose value during the 15 min preceding stimulation onset. Any trial in which mean pre-stimulus blood glucose was below 300 mg/dl was excluded. Time series data were then assessed in three windows of interest: pre-stimulation (15 min pre-stimulation), during stimulation (0–15 min post-stimulation onset), and post-stimulation cessation (0–120 min post-cessation). Within the post-stimulation cessation window, two periods were examined representative of various clinical interventions—a 15-min period analogous to short-acting insulin and a 120-min period analogous to long-acting insulin administration.

The rate of change in blood glucose was assumed to be linear within both 15-min and 2-h windows (Steil et al., 2011). A within window-of-interest rate of change in blood glucose was evaluated by fitting a linear regression to the time series data during the three different windows of interest.

A 95% confidence interval for blood glucose was generated based on pre-stimulation measurements. This identified a range of baseline blood glucose concentrations for each trial which was defined as having no change. Positive and negative blood glucose excursions were identified as each data point outside of the 95% pre-stimulus confidence interval. The total excursion duration was determined for each prescribed time window by summing the duration of each excursion within a trial. Finally, the area under the curve (AUC) for each excursion was calculated and summed to give a measure of the glycemic state during the excursion (Sakaguchi et al., 2016).

### Circulating hormone measurements

The endocrine response to stimulation was measured by sampling peripheral circulating blood. Prior to stimulation and 30 min after stimulation, 100 μl of blood was collected from the animal's tail vein into tubes containing 50 KIU of aprotinin (Figure 1A). The samples were allowed to clot for 30 min and were then centrifuged at 1,000 × *g* at 4°C for 15 min. The serum was collected and stored at −80°C until analysis. Insulin and glucagon were measured using an electrochemiluminescence assay according to the manufacturer's specifications (Meso-scale Discovery, Rockville, MD, USA). A *t*-test was used to determine the significance between stimulation and sham conditions, with a *p*-value of < 0.05 indicating significance.

### Laser speckle contrast imaging evaluation of pancreatic blood flow

Pancreatic perfusion in response to vagus nerve stimulation was evaluated using laser speckle contrast imaging (LSCI) in six animals. In brief, anesthesia was induced using isoflurane. The common carotid artery, jugular vein, and trachea were intubated for the purposes of blood pressure monitoring, i.v. infusion route, and facilitation of respiration, respectively. The body temperature was maintained at 37°C. The ventilatory state was monitored with an end-tidal pCO_2_ monitor, and the animals were artificially ventilated if necessary. A midline abdominal incision was made. A custom-made cuff electrode was implanted to ensure any changes in the neural interface were not reflected in this experiment. Due to the formation of adhesions preventing access to the pancreatic branch of the vagus nerve, this electrode is placed around the esophagus to stimulate the anterior trunk of the subdiaphragmatic vagus nerve rostral to the hepatic bifurcation. The duodenal lobe of the pancreas near the site of vagal insertion and the attached proximal segment of the duodenum were exteriorized and mounted on a plate for immobilization. The LSCI imager (Perimed PeriCam HR-PSI) was placed 10 cm above the mounting plate. Perfusion (blood flow) was continuously measured. Trains of up to 15 mA cathode-leading biphasic pulses for 800 μs/phase at 20 Hz were generated and delivered using a grass S88 square pulse stimulator and a PSIU-6 current-controlled stimulus isolator. Electrical stimulation of the pancreatic (*n* = 5) and/or subdiaphragmatic vagus nerve (*n* = 3) was applied for 3 s. At the end of the terminal experiment, the animals were euthanized and perfused with 4% paraformaldehyde.

### Histological assessment of islets

α- and β-cell populations were qualitatively evaluated using fluorescent immunohistochemistry. Tissue was collected from three groups: healthy animals (“control,” *n* = 2), STZ-induced animals (“diabetic,” *n* = 6), and STZ-induced with chronic pVNS (“diabetic pVNS,” *n* = 4). Paraformaldehyde-perfused pancreatic tissue from the duodenal lobe was frozen and sliced into 20 μm thick sections. The sections were blocked with 4% goat serum in Superblock (Thermo Scientific, Rockford, IL) for 1 h at room temperature. Following blocking, the sections were co-stained using mouse anti-glucagon (ab10988, Abcam, Cambridge, United Kingdom) and rabbit anti-insulin (RA20056, Neuromics, Edina, MN) at room temperature for 16 h. Both antibodies were diluted at 1:500 in 1% goat serum. The slices were washed with 1% goat serum in PBST four times for 15 min each. The sections were stained with Alexa Fluor 647 goat anti-rabbit IgG and Alexa Fluor 488 goat anti-mouse IgG (Invitrogen, Eugene, OR) for 3 h at room temperature followed by four additional washes. Images were acquired using a Keyence BZ-X700 (Keyence, Osaka, Japan) with a 20 × objective for islet morphology and cell type analysis using the same laser and capture settings between all samples. The pancreatic slice area was captured using a 4 × objective.

Islet morphological parameters were measured using custom scripting in ImageJ. In brief, islets were manually masked from each image to include any areas internal to the islet that were not insulin or glucagon positive. Insulin or glucagon-positive pixels were counted from each channel to determine protein expressing area. Area and perimeter were derived from each mask. The diameter was determined by using the major diameter of an ellipse fit to each mask. Islet density was determined by dividing the number of islets observed (healthy *n* = 253; diabetic *n* = 363; diabetic + pVNS *n* = 182) by the area of each slice.

### Statistical analysis

Statistical analysis was performed using Prism GraphPad 8.2.0. Changes in blood glucose were assessed by averaging trials within the subject and within the stimulus amplitude bin. A two-way ANOVA followed by Dunnett's test *post-hoc* was employed to identify differences between stimulus amplitudes. A one-way ANOVA with Tukey's *post-hoc* test was used for all histological analyses. For all statistical tests, a *p*-value of < 0.05 was used to determine significance.

## Results

### Electrical stimulation causes insulin secretion from β-cells

**Figure 2:**
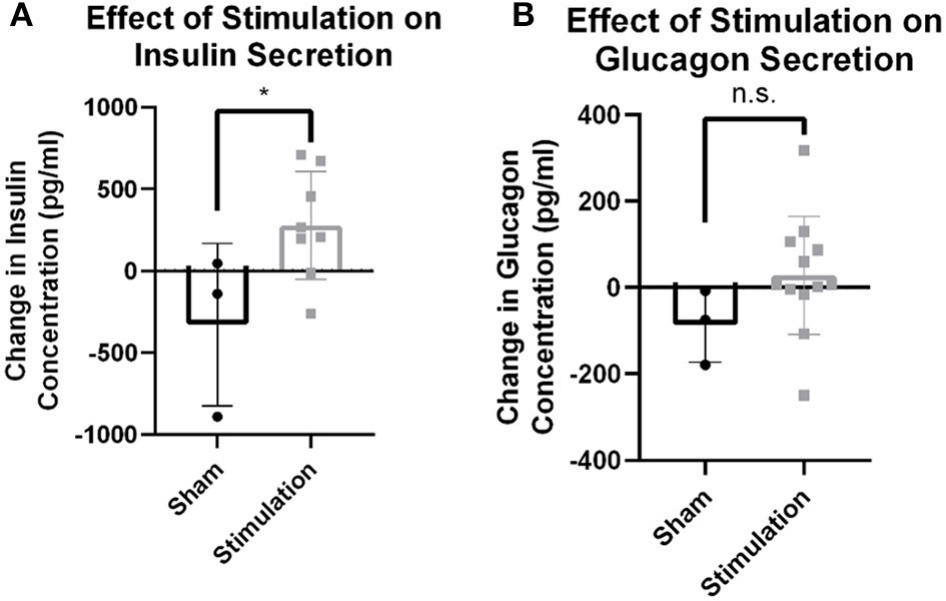
Hormone release from targeted vagus nerve stimulation using >0–300 μA. **(A)** Change in circulating insulin concentration after stimulation is increased compared to change due to sham stimulation as measured by tail vein blood draw (*p* = 0.0396). **(B)** Circulating glucagon concentration is not different between stimulation and sham conditions (*p* = 0.1922). All data were reported as mean ± standard deviation (sham: *n* = 3, 0–0.3 mA: *n* = 7). * is used to represent significant change relative to sham using *p* < 0.05.

Observed changes in blood glucose due to stimulation may either be the result of efferent drive to the islets resulting in endocrine secretion and subsequent glucose changes, or afferent signaling resulting in the activation of an unknown central reflex. To probe whether efferent affected β-cell or α-cell drive, serum concentrations of insulin and glucagon were assayed 30 min after stimulation onset. Stimulation amplitudes between 0 and 0.3 mA increased insulin levels detected in serum compared to sham stimulation (279.0 vs. −327.7 pg/ml, *p* = 0.04; Figure 2A). There was no statistically significant change observed in glucagon concentration after stimulation (*n* = 7) compared to the sham (*n* = 3; 28.2 vs. −87.2, *p* = 0.13; Figure 2B). This suggests that stimulation may result in direct β-cell drive and that this stimulation does not robustly activate α-cells.

### Parasympathetic neuromodulation does not change pancreatic blood flow

**Figure 3:**
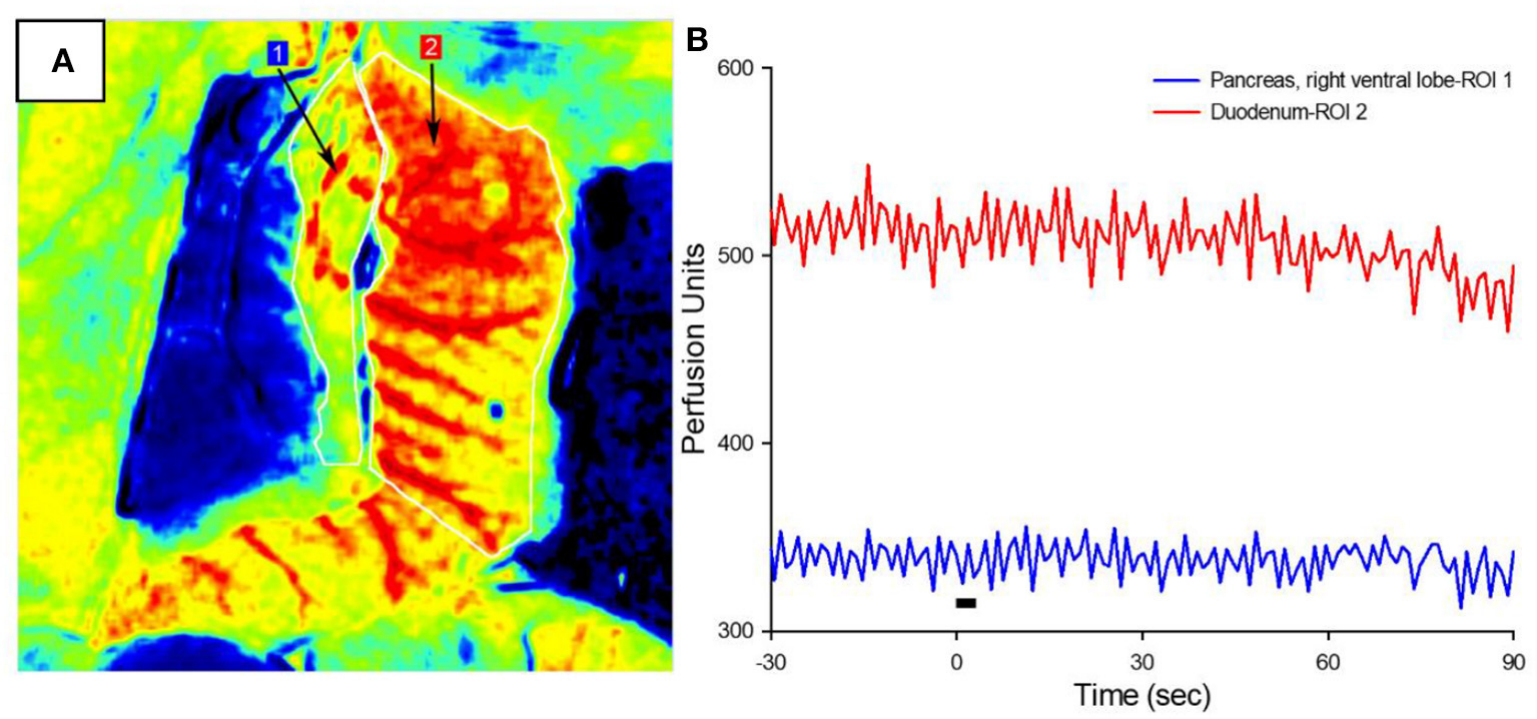
Pancreatic blood flow during targeted vagus nerve stimulation. **(A)** Representative LSCI image of the pancreas (ROI #1) and duodenum in response to subdiaphragmatic vagus nerve stimulation. **(B)** Perfusion as a function of time before, during, and after 3-s VNS (denoted by the black bar). Note that vagal stimulation did not produce a change in pancreatic blood flow.

The increased circulating insulin levels may be the result of either the direct β-cell release of insulin or the increased organ perfusion from parasympathetic-induced vasodilation. To determine the cause of this increased insulin concentration, blood flow in the right ventral (duodenal) lobe of the pancreas and attached proximal duodenum was measured via LSCI in response to the parasympathetic neuromodulation of the subdiaphragmatic vagal trunks (Figure 3A). A 3-s train of 10 mA, 0.8 ms pulse duration at 20 Hz was applied to the subdiaphragmatic vagus nerve. Blood flow values over time (measured in perfusion units) showed the lack of immediate pancreatic blood flow changes with VNS (Figure 3B) in all six diabetic animals with implants. Stimulation of the subdiaphragmatic vagus nerve rostral to the hepatic bifurcation also resulted in no change in immediate pancreatic blood flow (*n* = 3). Similarly, pancreatic blood flow did not change in diabetic rats without implants or non-diabetic controls (*n* = 5). It is important to note that in all animals, decreased pancreatic blood flow (vasoconstriction) was produced by the electrical stimulation of the sympathetic fibers in the left splanchnic nerve (Kundu et al., 2019) as a test of blood flow measurement validity. Although vagal stimulation produced a small and delayed increase in blood pressure, this did not produce an increase in pancreatic blood flow. These results confirm that the subdiaphragmatic VNS stimulation did not produce a change in pancreatic blood flow and suggest that insulin changes are not the result of changes in transport based on perfusion rate changes.

### Pre-stimulation blood glucose is not dependent on prior or subsequent stimulation

**Figure 4:**
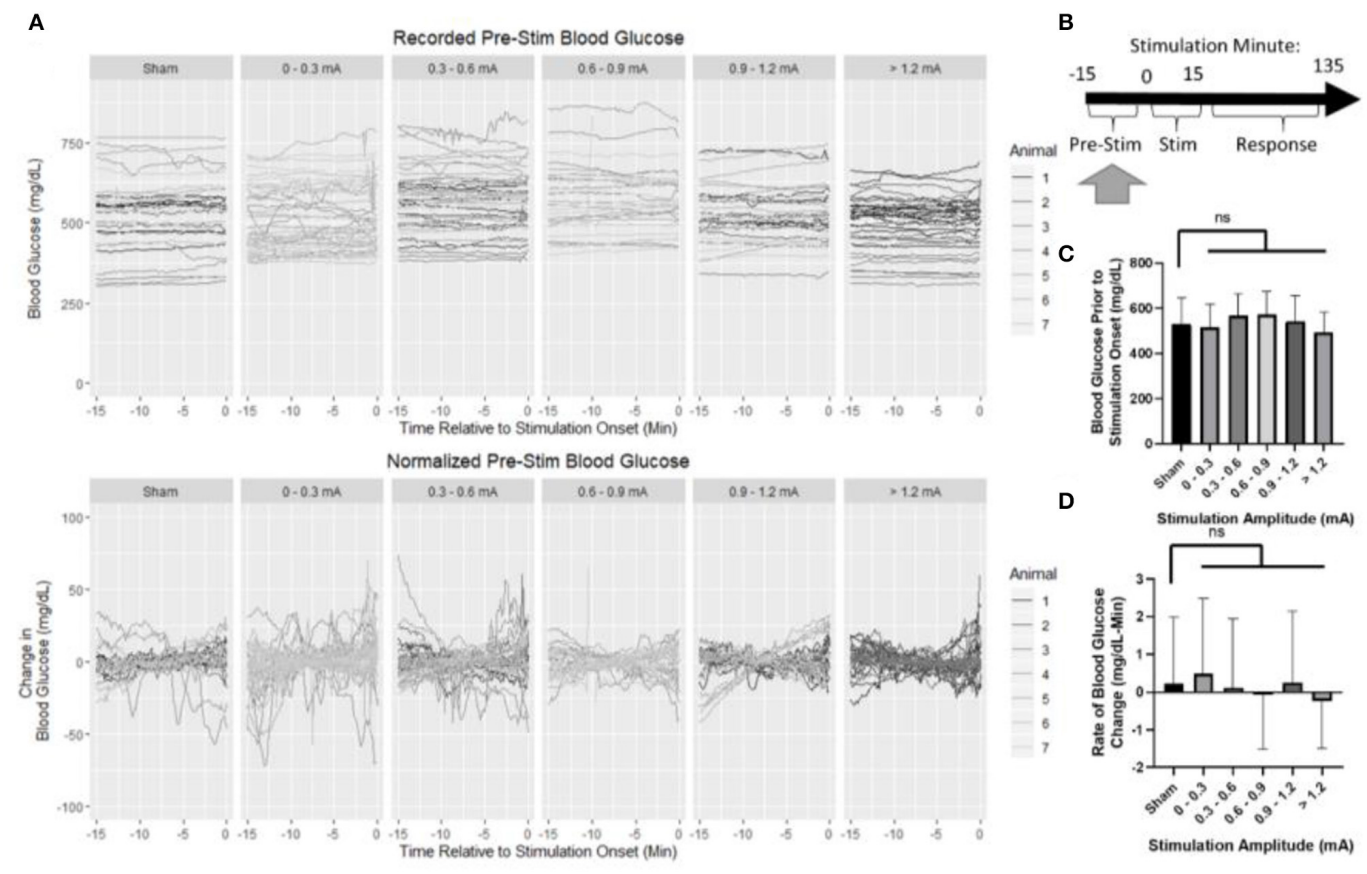
Blood glucose prior to the onset of stimulation. **(A)** Recorded and normalized blood glucose show similar distributions throughout the dataset. **(B)** Timeline showing where data were collected for this analysis. **(C)** Mean blood glucose was not different between sham and any stimulation bin. **(D)** Rate of change in blood glucose was not different between sham and any stimulation bin. All data reported as mean ± standard deviation (sham: *n* = 42, 0–0.3 mA: *n* = 79, 0.3–0.6 mA: *n* = 52, 0.6–0.9 mA: *n* = 35, 0.9–1.2 mA: *n* = 42, >1.2 mA: n = 36).

Baseline blood glucose was first assessed to ensure that normalization to pre-stimulus measurements was appropriate (Figure 4A). The normalization period consisted of 15 min prior to the onset of stimulation (Figure 4B). Mean blood glucose prior to sham stimulation (530 mg/dl) was compared to each stimulation amplitude (Figure 4C) and not found to be statistically different from 0 to 0.3 mA (518 mg/dl, *p* = 0.98), 0.3 to 0.6 mA (567 mg/dl, *p* = 0.36), 0.6 to 0.9 mA (572 mg/dl, *p* = 0.31), 0.9 to 1.2 mA (541 mg/dl, *p* = 0.98), or >1.2 mA (493 mg/dl, *p* = 0.47).

The rate of change in blood glucose was determined by linear regression to assess stationarity (Figure 4D). No significant difference was observed between sham (0.22 mg/dl -min) and 0–0.3 mA (0.49 mg/dl-min, *p* = 0.95), 0.3–0.6 mA (0.1 mg/dl-min, *p* = 0.99), 0.6–0.9 mA (−0.08 mg/dl-min, *p* = 0.93), 0.9–1.2 mA (0.25 mg/dl-min, *p* = 0.999), or >1.2 mA (−0.24 mg/dl-min, *p* = 0.70). Together, these data suggest that prior to stimulation, each group is similar, and normalization of each trial to its pre-stimulation mean blood glucose concentration is appropriate.

### Blood glucose effects during stimulation

**Figure 5:**
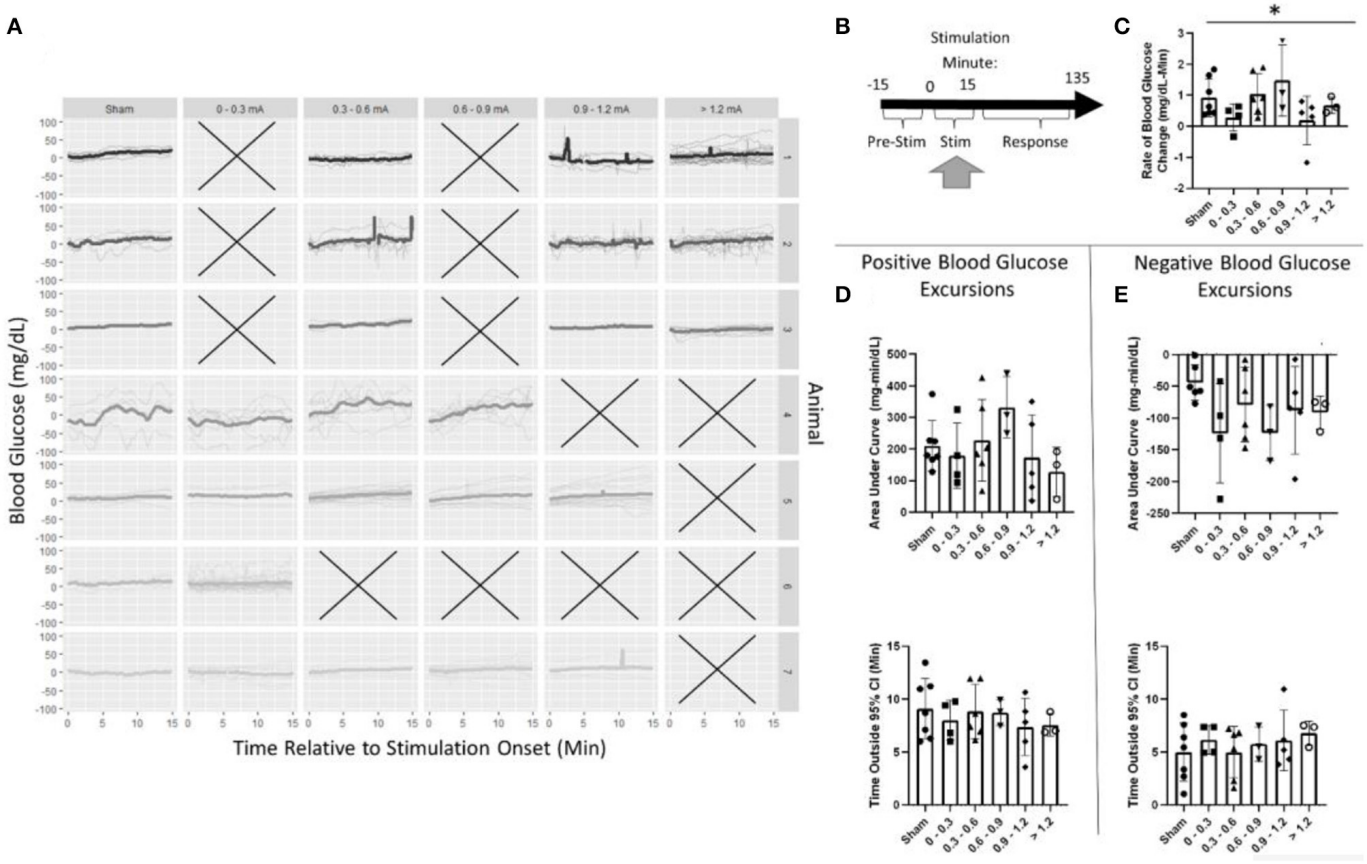
Blood glucose during stimulation. **(A)** Individual trials and normalized blood glucose for each stimulation bin within an animal. **(B)** Timeline showing where data were collected for this analysis. **(C)** A two-way ANOVA determined that stimulation amplitude was a significant predictor for the rate of change in blood glucose; however, no changes between sham and any group were observed using *post-hoc* testing. **(D)** Assessment of the area under the curve **(top)** and duration **(bottom)** of positive blood glucose excursions from the 95% CI showed no significant differences. **(E)** Assessment of the area under the curve **(top)** and duration **(bottom)** of negative blood glucose excursions from the 95% CI showed no significant differences. All data reported as mean ± standard deviation (sham: *n* = 7, 0–0.3 mA: *n* = 4, 0.3–0.6 mA: *n* = 6, 0.6–0.9 mA: *n* = 3, 0.9–1.2 mA: *n* = 5, >1.2 mA: *n* = 3). *p < 0.05.

The effects of pancreatic neuromodulation on the change in blood glucose were examined while the stimulation was applied (Figure 5B). Due to the *post-hoc* calibration of the stimulation, no data were collected for some stimulation conditions in each animal as denoted by an X (Figure 5A). Generally, across all groups (including sham), blood glucose concentration increased during targeted stimulation compared to baseline measurements (Figure 5A). During the 15-min stimulation window, blood glucose increased at a rate of 0.9 mg/dl-min for sham-stimulated rats. A two-way ANOVA found stimulation amplitude to be a significant predictor of blood glucose rate of change (*p* = 0.039). However, the *post-hoc* analysis did not find a significant difference between any of the individual stimulation amplitudes and sham (Figure 5C, Supplementary Table 1). A two-way ANOVA also determined animals to be a significant predictor of blood glucose slope, positive AUC, and negative AUC (Supplementary Table 1). These results suggest that pVNS does affect the concentration of blood glucose in a stimulus amplitude-dependent manner; however, no specific amplitude was identified as having a significantly different effect possibly suggesting an underpowered study.

During stimulation, the positive AUC for sham stimulation was 210 mg/dl-min. This was not significantly different from any stimulation amplitude (Figure 5D, Supplementary Table 1). Mean blood glucose was elevated for 9.1 min in sham-stimulated animals and was not significantly different from any stimulation amplitude (Figure 5D, Supplementary Table 1). Similarly, neither negative AUC nor the duration of the excursion was different between sham stimulation and any stimulation amplitude (Figure 5E, Supplementary Table 1).

### Blood glucose effects after stimulation

**Figure 6:**
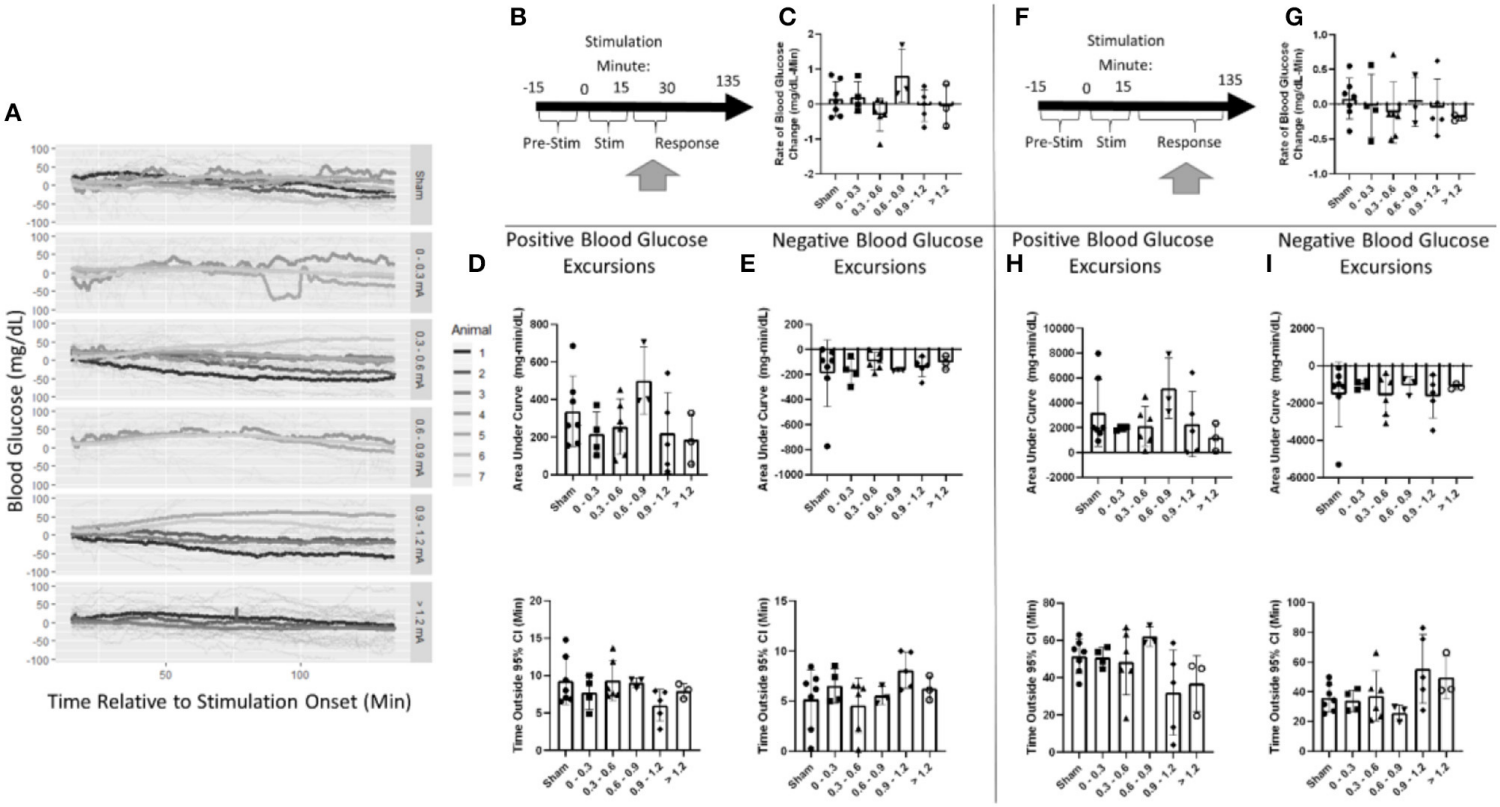
Blood glucose post-stimulation cessation. **(A)** Mean blood glucose values for each animal within a stimulation amplitude. **(B)** Timeline showing where data were collected for the 15-min post-stimulation analysis. **(C)** Stimulation amplitude was not found to be a significant factor for the rate of change in blood glucose after stimulation. **(D)** Assessment of the area under the curve **(top)** and duration **(bottom)** of positive blood glucose excursions from the 95% CI showed no significant differences. **(E)** Assessment of the area under the curve **(top)** and duration **(bottom)** of negative blood glucose excursions from the 95% CI showed no significant differences. **(F)** Timeline showing where data were collected for the 120-min post-stimulation analysis. **(G)** Stimulation amplitude was not found to be a significant factor for the rate of change in blood glucose after stimulation. **(H)** Assessment of the area under the curve **(top)** and duration **(bottom)** of positive blood glucose excursions from the 95% CI showed no significant differences. **(I)** Assessment of the area under the curve **(top)** and duration **(bottom)** of negative blood glucose excursions from the 95% CI showed no significant differences. Blood glucose during the application of targeted vagus nerve stimulation (sham: *n* = 7, 0–0.3 mA: *n* = 4, 0.3–0.6 mA: *n* = 6, 0.6–0.9 mA: *n* = 3, 0.9–1.2 mA: *n* = 5, >1.2 mA: *n* = 3).

Post-stimulation, blood glucose was evaluated in two windows (Figure 6A). First, a 15-min window immediately after stimulation the cessation was assessed (Figure 6B, Supplementary Table 2). Blood glucose increased at a rate of 0.2 mg/dl-min in the sham stimulation group This was not found to be significantly different from any stimulation amplitude (Figure 6C, Supplementary Table 2).

Blood glucose excursions outside of the 95% confidence interval calculated from pre-stimulation blood glucose were evaluated to determine a change in the glycemic state. After the cessation of stimulation, a positive AUC for the sham was 337 mg/dl-min (Figure 6D). Mean blood glucose was elevated for 9.3 min in sham-stimulated animals (Figure 6D). No significant difference in either AUC or duration in positive excursions was observed (Supplementary Table 2). The negative AUC for sham stimulation was −191 mg/dl, and mean blood glucose was decreased for 5.2 min in sham-stimulated animals (Figure 6E). Neither of these parameters was significantly influenced by any stimulation amplitude (Supplementary Table 2). A two-way ANOVA determined a significant effect between animals (Supplementary Table 2). This suggests that animals respond differently to the same stimulation. Due to the required *post-hoc* recalibration of stimulus amplitude, a full-factorial dataset does not exist to further probe this interaction.

Blood glucose was also evaluated for long-term changes due to stimulation during a 120-min window following the cessation of stimulation (Figure 6F). Overall, blood glucose was relatively stable changing at a rate of 0.1 mg/dl-min in the sham stimulation group (Figure 6G). This was not found to be significantly different from any stimulation amplitude (Supplementary Table 3). The positive AUC for sham was 3,192 mg/dl-min (Figure 6H). Mean blood glucose was elevated for 51.4 min in sham-stimulated animals (Figure 6H). Neither of these measures was changed due to any stimulation amplitude (Supplementary Table 3). Negative AUC was −1,540 mg/dl and was decreased for 36.1 min in sham-stimulated animals (Figure 6I). This was not significantly different from any stimulation (Supplementary Table 3). These data suggest that there is no effect observed after the cessation of stimulation in both immediate and long-term windows.

### Histological assessment of chronically stimulated pancreas

**Figure 7:**
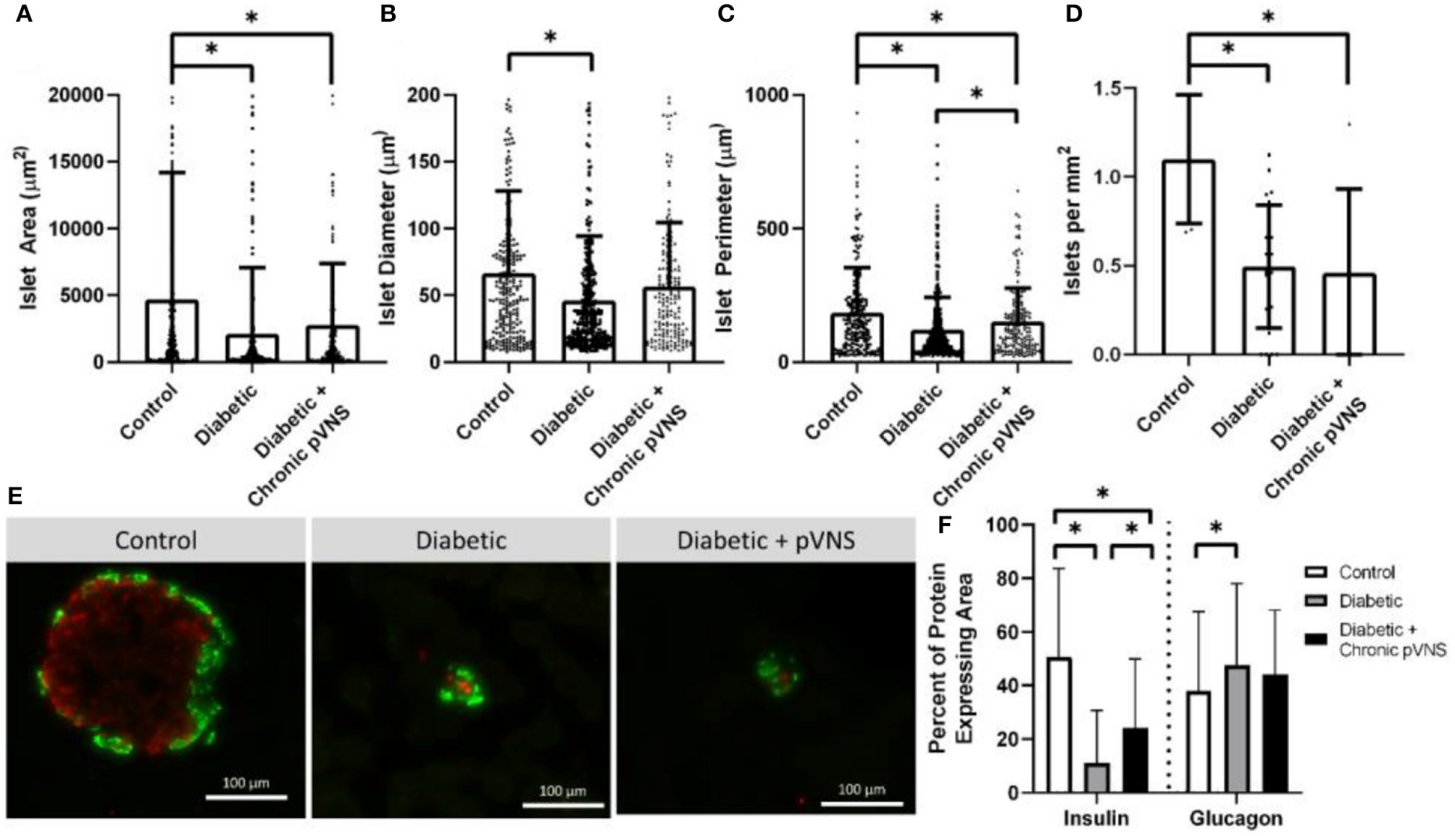
Effect of pVNS on islet morphology. **(A)** Both diabetic and pVNS islets have a smaller cross-sectional area than healthy islets. **(B)** Islet diameter is smaller in diabetic islets but not pVNS islets. **(C)** Islet perimeter is decreased in both diabetic and pVNS islets; however, pVNS is larger than the diabetic perimeter. **(D)** Islet density is lower in both diabetic and pVNS groups compared to healthy. **(E)** Representative islets from each group show insulin-positive β-cells (red) and glucagon-positive α-cells (green). **(F)** Diabetic and pVNS islets show decreased insulin-positive area; however, pVNS has an elevated insulin-positive area compared to diabetic without stimulation. Only diabetic without stimulation shows an increase in glucagon-positive area. All data were reported as mean ± standard deviation (sham: *n* = 2, diabetic: *n* = 6, diabetic + pVNS: *n* = 4). * is used to represent significance using a *p*-value of < 0.05.

After terminal blood flow experiments, the duodenal lobe of the pancreas was examined for changes due to receiving chronic random stimulation. This experiment was designed to test if receiving any pVNS resulted in changes in islet cell populations (Figure 7E). It is important to note that not all subjects received the same amount of charge injected for the chronic stimulation study.

Islets were decreased in size according to all metrics after STZ induction relative to healthy counterparts. Healthy islets (4,693 μm^2^) had larger cross-sectional area than diabetic islets (2,104 μm^2^, *p* < 0.001) and diabetic islets that received chronic pVNS (2,771 μm^2^, *p* = 0.009); there was no difference observed between diabetic and diabetic pVNS groups (*p* = 0.51; Figure 7A). The mean diameter of healthy islets (66.4 μm) was larger than diabetic islets (46.0 μm, *p* < 0.001); however, this was not observed in diabetic pVNS islets (56.4 μm, *p* = 0.13; Figure 7B). Healthy islets (4,693 μm^2^) had larger cross-sectional area than diabetic islets (2,104 μm^2^, *p* < 0.001) and diabetic islets that received chronic pVNS (2,771 μm^2^, *p* = 0.009; Figure 7C) Diabetic pVNS islets had a larger perimeter than diabetic islets that did not receive pVNS (*p* = 0.497). To assess whether pVNS had any protective or restorative effects on islets, the number of islets per mm^2^ of tissue was calculated. Healthy tissue contained 1.1 islets/mm^2^, a higher concentration of islets than either diabetic (0.5 islets/mm^2^, *p* = 0.002) or diabetic pVNS pancreas (0.5 islets/mm^2^, *p* = 0.002; Figure 7D).

A histological analysis was used to examine whether pVNS affected cell populations within the islet. As expected after STZ treatment, the insulin-expressing area in diabetic (11.8% *p* < 0.001) and diabetic + pVNS (24.2%, *p* < 0.001) islets was smaller than healthy control islets (39.4%). Diabetic pVNS islets did have an elevated insulin-positive area compared to diabetic islets (*p* < 0.001). Glucagon-expressing area was increased from healthy (38.0%) in diabetic (47.6% *p* < 0.001; Figure 7F) but not diabetic pVNS conditions (44.1%, *p* < 0.06). Diabetic pVNS islets did not have a different glucagon-positive area compared to diabetic islets (*p* < 0.4; Figure 7F).

## Discussion

In this study, we present data demonstrating the effects of targeted pancreatic neuromodulation by stimulating a branch of the vagus nerve just prior to pancreatic insertion. Previously, untargeted parasympathetic neuromodulation of the pancreas has been investigated using cervical or subdiaphragmatic vagus nerve stimulation (Daniel and Henderson, 1967; Frohman et al., 1967; Kaneto et al., 1967; Kundu et al., 2019; Yin et al., 2019). The untargeted approach results in the activation of fibers innervating the stomach, small intestine, liver, as well as pancreas—all organs whose function may perturb blood glucose homeostasis. Without anatomical targeting of electrical stimulation toward a specific organ, the activation of numerous physiological processes may interact in an additive or opposing manner when considering effects on blood glucose. This has been previously demonstrated. For example, Meyers et al. (2016) further found that the stimulation of the intact cervical nerve resulted in hyperglycemia without an increase in insulin. Untargeted stimulation obfuscates mechanisms and has led to the publication of datasets that contradict each other. The targeted stimulation used in this study aimed to minimize off-target effects.

The study presented here investigates the effects of pVNS on blood glucose and pancreatic morphology using a model of T1D. Many previous studies have been conducted primarily in either healthy or type 2 diabetic models (Yin et al., 2019). The findings of our study may have more relevance for T1D patients.

### Targeted neuromodulation of blood glucose

While the complete mechanistic effect of blood glucose modulation via cervical VNS is unclear, it is consistent throughout the literature that the stimulation of the vagus nerve modulates blood glucose concentration through efferent signaling and peripheral modulation of β-cell function (Daniel and Henderson, 1967; Frohman et al., 1967; Kaneto et al., 1967; Nelson et al., 1967). This is accomplished when acetylcholine released from the fibers of the vagus nerve activates the pancreatic β-cells expressing M_3_ muscarinic receptors. This signaling cascade results in a host of downstream responses including the activation of PLC, PLA_2_, and PLD. Together this increases PKC activation, a critical mediator of calcium and insulin exocytosis.

Based on this mechanism, the β-cell release of insulin still relies on a high concentration of cytosolic calcium for exocytosis. High levels of intracellular calcium must precede stimulation and are primarily increased during physiological hyperglycemia. Therefore, pVNS-induced insulin release is only effective during hyperglycemic states. The data we collected (not shown) have supported that there is no decrease in blood glucose concentration during normoglycemic stimulation and provides a rationale for excluding trials with an initial blood glucose level below 300 mg/dl, the predefined cutoff for STZ-diabetic induction.

Pancreatic neuromodulation could offer high-precision blood glucose control that is temporally specific with a titratable magnitude. Our data suggest that the modulation of blood glucose kinetics occurs during the 15-min stimulation session. While blood glucose was not significantly different in any of the groups, stimulation amplitude was found to be a predictor of blood glucose rate of change. Furthermore, increased circulating insulin was detected after stimulation. These effects do not appear to continue after stimulation has ended; stimulation amplitude was not found to be a predictor of blood glucose concentration for either post-stimulation time period tested. This suggests that pancreatic neuromodulation offers control over blood glucose with high temporal specificity. This is likely due to a combination of exhausting the limited amount of insulin and the rapid breakdown of acetylcholine after release. Additionally, the data show that stimulation amplitude influences the magnitude of the change in blood glucose kinetics which demonstrates the potential for a titratable response. This non-linear effect may be due to the innervation of competing α- and β-cells. Stimulation may simultaneously cause the release of insulin and glucagon which have countering roles in glucose homeostasis. Another possible explanation is the innervation of pancreatic ganglia by the vagus nerve. Although these innervation patterns have not been fully investigated, integration of sympathetic and parasympathetic signaling in these ganglia may override the direct effects of β-cells' innervation by the vagus.

While statistical analysis identified stimulation as being a significant predictor of blood glucose change, a *post-hoc* analysis did not determine any specific amplitude as being significantly different than the sham. This may be due to a combination of factors that impact the ability to assess amplitude response in a small-sized cohort. The *post-hoc* analysis identified the subject as being a significant predictor of the effect of the kinetics of blood glucose change and measures of AUC suggesting a responder effect. This is likely due to a combination of factors including variable disease state, anatomical differences in innervation patterns, stability of neural interface, or neural damage during/after electrode implantation. Human-prescribed therapies agree with this proposition; clinicians must tune stimulation parameters for each patient. Labar observed a range of patient-dependent cervical VNS efficacy between 0.25 and 2 mA in reducing seizures (Nelson et al., 1967). Additionally, the effect of VNS is dependent not only on stimulation amplitude but also on stimulation frequency and pulse width (Labar, 2004). The data reported here do not investigate the effect of these parameters; thus, the chosen parameters may not be the most appropriate for therapeutic pancreatic neuromodulation in each animal.

Electrodes implanted in some animals had high impedance and prevented the delivery of exact stimulation amplitude due to stimulator compliance. To compensate for this, the delivered current was corrected *post-hoc*, and the continuous range of amplitudes was binned into discrete stimulation widows. This leads to a phenomenon where the data represent average responses to a stimulus range instead of a specific treatment. As a result, the data indicate that the study was underpowered. Due to the low number of implanted animals, this analysis was unavoidable but may hide small resolution effects.

It cannot be ruled out that the implantation of the cuff electrode on the small diameter pancreatic branch of the vagus nerve directly affected the viability of the nerve. A previous cohort of animals in this study (*n* = 12) was implanted with shape memory polymer electrodes. These devices softened when warmed by body temperature in an effort to minimize mechanical compliance mismatch between the device and the tissue. Unfortunately, device stability was insufficient for the required duration of this study, and no modulation of blood glucose data could be collected. Furthermore, it cannot be ruled out that the dissection of the small diameter nerve damaged some of the few fibers within the nerve which could lead to a significant percentage of fibers being affected.

### Possible mechanism: insulin secretion from residual β-cells

Our data suggest that the stimulation of the pancreatic branch of the vagus nerve results in a modest increase in circulating insulin concentration 30 min after the onset (15 min after the end) of stimulation. The magnitude of this release had a mean value of 279 pg/ml. This is on the same order of magnitude of insulin release that has been reported in the acute efferent stimulation of the vagus nerve in rodents (Meyers et al., 2016). While glucose and insulin regulation in rats is vastly different between rodents and non-human primates, the observed increase is one order of magnitude smaller than what has been previously reported (Kundu et al., 2019). These results in the context of our setup are promising and suggest that neuromodulation may have been used as a therapy even in a T1D-limited β-cell model. Furthermore, temporal aspects of our data suggest that insulin release is likely due to the direct drive of β-cells, rather than secondary effects of off-target or afferent stimulation.

The transport of insulin from the islets to the body is reliant on organ perfusion. The vagus nerve not only innervates the β-cells but also vasculature around the cells. Parasympathetic drive to blood vessels results in vasodilation, thereby increasing the amount of blood flow through an organ. Our LSCI data support previous reports that parasympathetic signaling is not the major neural modulator of blood flow to the pancreas (Rodriguez-Diaz et al., 2011). This importantly demonstrates that the increased amount of insulin that was detected following stimulation was the result of modulated parasympathetic drive to the β-cells rather than an increased export rate.

### Effects of chronic pVNS on pancreatic tissue

In this study, we used a chemically induced model of T1D in which STZ results in rapid β-cell death due to DNA methylation. As evaluated histologically, islet health decreased by multiple quantitative measures in STZ-treated tissue compared to healthy tissue. Animals that did not receive stimulation had decreased islet area, diameter, perimeter, and fewer islets/mm^2^. The islets had a smaller proportion of insulin-positive area and a larger proportion of glucagon-positive area demonstrating β-cell death.

Islet cell populations were probed to examine the effect of chronic pancreatic neuromodulation on islet health. As expected from STZ induction, the islet cross-sectional area had decreased, the islets had a smaller perimeter, and there were fewer islets/ mm^2^. Interestingly, animals that received stimulation had a significantly larger islet perimeter than STZ animals. Additionally, islet diameter was not found to be decreased in the healthy controls. and α-cell hyper-proliferation that has been observed in the STZ model was not observed in the islets that received chronic pVNS (Hulsey et al., 2017). Finally, the islets that received chronic pVNS had a larger percentage of insulin-positive area than those that did not, though not as great as healthy controls.

In this set of experiments, stimulation was applied after the onset and confirmation of STZ-induced T1D. Due to the short half-life and rapid excretion of STZ, by the time the stimulation had commenced, STZ-induced b-cell destruction would have been completed. This model does not consider the effects of the chronic autoimmune attack that is seen in human T1D. As such, any protective effect toward β-cell health cannot be extrapolated to what is observed in T1D. The slight increase in histological measures observed that chronic pVNS may influence islet macrostructure even when there is no autoimmune attack. The observed trends toward restoration from a disease state across multiple histological measures do warrant further investigation.

## Conclusion and implications for the treatment of T1D

While the data presented within this study are promising, the context and constraints under which these data were collected must be considered. These data were only collected during a snapshot of this disease. T1D is a progressive disease in which an autoimmune attack continually destroys the β-cells. Symptoms of disease onset are often not observed until approximately 90% loss in the β-cell mass (Zhang et al., 2012). Contrary to the autoimmune disease, the STZ-induced T1D model does not have a progressive loss of β-cells but instead quickly results in the large-scale destruction of β-cells leading to hyperglycemia. As such, these complications of progressive β-cell are not accurately captured.

Islet structure and innervation patterns differ between rodents and humans. As such, stimulation parameters that affect blood glucose in rodents are not necessarily similar to the parameters that may be needed for effective human neuromodulation therapies. The importance of these findings suggests that targeted neuromodulation may influence systemic levels of blood glucose in a diseased pancreas which has relevance in developing future T1D therapies. However, additional work needs to be carried out in models that more closely mimic human anatomy and disease progression to better investigate the feasibility of targeted neuromodulation as a therapy.

Throughout the duration of the study, animals remained dependent on daily insulin. As such, it is clear that pVNS did not cure or reverse the T1D state induced by STZ. Data presented here show that pVNS should be investigated in a large animal model by focusing on blood glucose modulation during the delivery of stimulation as opposed to after the cessation of stimulation. Future studies in a more clinically relevant model can help elucidate the therapeutic potential in humans.

## Data availability statement

The raw data supporting the conclusions of this article will be made available by the authors, without undue reservation.

## Ethics statement

The animal study was reviewed and approved by University of Florida Institutional Animal Care and Use Committee (IACUC) as well as the Animal Care and Use Review Office (ACURO).

## Author contributions

Data analysis was conducted by ED. All authors contributed to the experimental design, data collection, manuscript generation, and editing.
