# Supplementary material for: The effects of targeted vagus nerve stimulation on glucose homeostasis in STZ-induced diabetic rodents
Source: Front Neurosci. 2023 Jun 15;17:1179276. doi: 10.3389/fnins.2023.1179276 (PMC10309008; doi:10.3389/fnins.2023.1179276)
Supplement: Supplementary file 1 [file Data_Sheet_1.docx]

Supplementary Table 1: Statistical Analysis of pVNS modulated blood glucose during stimulation. Stimulation amplitude was determined to be a significant predictor for the rate of change in blood glucose while stimulus was applied. It was also observed that the categorical variable assigned to each animal was significant in predicting the slop as well as both positive and negative AUC.

|  |  | **During Stim**  **Slope** | | **During Stim**  **Positive AUC** | | **During Stim**  **Positive Time** | | **During Stim**  **Negative AUC** | | **During Stim**  **Negative Time** | |
| --- | --- | --- | --- | --- | --- | --- | --- | --- | --- | --- | --- |
|  | n | Mean  (mg/dL-min) | *p* | Mean  (mg-min/dL) | *p* | Mean  (min) | *p* | Mean  (mg-min/dL) | *p* | Mean  (min) | *p* |
| **Stimulation** | - | - | **0.039** | - | 0.11 | - | 0.832 | - | 0.884 | - | 0.807 |
| **Animal** | - | - | **0.043** | - | **0.026** | - | 0.642 | - | **0.010** | - | 0.320 |
| **Sham** | 7 | 0.92 ± 0.61 | - | 211 ± 79.6 | - | 9.1 ± 2.9 | - | -99 ± 146 | - | 5.0 ± 2.7 | - |
| **0-0.3 mA** | 4 | 0.28 ± 0.43 | 0.488 | 179 ± 103 | - | 8.0 ± 1.9 | - | -125 ± 77.9 | - | 6.2 ± 1.4 | - |
| **0.3-0.6 mA** | 6 | 1.1 ± 0.66 | 0.998 | 228 ± 129 | - | 8.8 ± 2.6 | - | -79 ± 58.6 | - | 5.0 ± 2.4 | - |
| **0.6-0.9 mA** | 3 | 1.5 ± 1.2 | 0.692 | 332 ± 97.2 |  | 8.7 ± 1.2 | - | -124 ± 42.9 | - | 5.8 ± 1.6 | - |
| **0.9-1.2 mA** | 5 | 0.20 ± 0.79 | 0.297 | 173 ± 135 | - | 7.4 ± 2.7 | - | -88 ± 69 | - | 6.1 ± 2.9 | - |
| **> 1.2 mA** | 3 | 0.67 ± 0.26 | 0.982 | 128 ± 77.9 | - | 7.6 ± 1.1 | - | -91.4 ± 26.2 | - | 6.8 ± 1.2 | - |

Supplementary Table 2: Statistical Analysis of pVNS modulated blood glucose during the 15-minute period after cessation of stimulation. The only significant factor observed was the categorical variable assigned per animal when predicting negative AUC.

|  |  | **After Stim**  **Slope** | | **After Stim**  **Positive AUC** | | **After Stim**  **Positive Time** | | **After Stim**  **Negative AUC** | | **After Stim**  **Negative Time** | |
| --- | --- | --- | --- | --- | --- | --- | --- | --- | --- | --- | --- |
|  | n | Mean  (mg/dL-min) | *p* | Mean  (mg-min/dL) | *p* | Mean  (min) | *p* | Mean  (mg-min/dL) | *p* | Mean  (min) | *p* |
| **Stimulation** | - | - | 0.105 | - | 0.131 | - | 0.285 | - | 0.7661 | - | 0.232 |
| **Animal** | - |  | 0.349 |  | 0.130 | - | 0.511 | - | **0.0461** | - | 0.448 |
| **Sham** | 7 | 0.15 ± 0.49 | - | 337 ± 186 | - | 9.3 ± 3.2 | - | -191 ± 267 | - | 5.2 ± 2.9 | - |
| **0-0.3 mA** | 4 | 0.20 ± 0.43 | 0.488 | 215 ± 118 | - | 7.7 ± 2.3 | - | -178 ± 100 | - | 6.5 ± 1.7 | - |
| **0.3-0.6 mA** | 6 | -0.31 ± 0.46 | 0.998 | 255 ± 147 | - | 9.4 ± 2.7 | - | -96 ± 71 | - | 4.6 ± 2.7 | - |
| **0.6-0.9 mA** | 3 | 0.81 ± 0.76 | 0.692 | 500 ± 179 | - | 9.1 ± 0.7 | - | -166 ± 4 | - | 5.6 ± 0.9 | - |
| **0.9-1.2 mA** | 5 | -0.05 ± 0.45 | 0.298 | 221 ± 216 | - | 6.0 ± 2.1 | - | -142 ± 78 | - | 8.1 ± 1.8 | - |
| **> 1.2 mA** | 3 | -0.06 ± 0.61 | 0.982 | 186 ± 136 | - | 8.0 ± 1.0 | - | -105 ± 53 | - | 6.3 ± 1.2 | - |

Supplementary Table 3: Statistical Analysis of pVNS modulated blood glucose for the 120-minute period after cessation of stimulation. No significant differences are observed in the two-hour period after stimulation.

|  |  | **After Stim**  **Slope** | | **After Stim**  **Positive AUC** | | **After Stim**  **Positive Time** | | **After Stim**  **Negative AUC** | | **After Stim**  **Negative Time** | |
| --- | --- | --- | --- | --- | --- | --- | --- | --- | --- | --- | --- |
|  | n | Mean  (mg/dL-min) | *p* | Mean  (mg-min/dL) | *p* | Mean  (min) | *p* | Mean  (mg-min/dL) | *p* | Mean  (min) | *p* |
| **Stimulation** | - | - | 0.785 | - | 0.203 | - | 0.141 | - | 0.915 | - | 0.129 |
| **Animal** | - | - | 0.051 | - | 0.071 | - | 0.324 | - | 0.334 | - | 0.364 |
| **Sham** | 7 | 0.08±0.29 | - | 4577±3792 | - | 65±10.5 | - | -2313±2330 | - | 52±11 | - |
| **0-0.3 mA** | 4 | -0.02±0.45 | 0.488 | 2857±599 | - | 66±9.4 | - | -1734±586 | - | 48±11 | - |
| **0.3-0.6 mA** | 6 | -0.12±0.44 | 0.998 | 3097±2622 | - | 61±24 | - | -2581±1652 | - | 54±23 | - |
| **0.6-0.9 mA** | 3 | 0.03±0.35 | 0.692 | 6915±3637 |  | 78±6.2 | - | -1776±722 | - | 40±6 | - |
| **0.9-1.2 mA** | 5 | -0.05±0.41 | 0.298 | 3276±3859 | - | 42±30 | - | -2562±1702 | - | 76±31 | - |
| **> 1.2 mA** | 3 | -0.20±0.04 | 0.982 | 1494±1329 | - | 47±20 | - | -1746±224 | - | 69±20 | - |
